# Molecular identification of two entomopathogenic fungus *Clonostachys rosea* strains and their efficacy against two aphid species in Iraq

**DOI:** 10.1186/s43141-022-00347-y

**Published:** 2022-04-28

**Authors:** Akram A. Mohammed, Firas A. Ahmed, Amal S. Younus, Ali A. Kareem, Ali M. Salman

**Affiliations:** 1 Plant Protection Department, University of Kufa, Najaf, Iraq; 2 Al-Najaf Directorate of Agriculture, Ministry of Agriculture, Najaf, Iraq; 3 Plant Protection Department, University of Karbala, Karbala, Iraq; 4 School of Environment and Rural Science, University of New England, Armidale, Australia

**Keywords:** *Myzus persicae*, *Aphis fabae*, Phylogenetic analysis, Virulence, Biological control

## Abstract

**Background:**

The green peach aphid, *Myzus persicae* Sulzer, and the bean aphid, *Aphis fabae* Scopoli (both Hemiptera: Aphididae), are serious pests of greenhouse vegetable crops in Iraq and other regions of the globe. In this study, two morphological identical isolates (AA80 and AA82) of the entomopathogenic fungus *Clonostachys rosea* Schroers (Hypocreales: Bionectriaceae) from Iraq were isolated and characterized with phylogenetic analysis based on the internal transcribed spacer (ITS) region. The efficacy of *C. rosea* against *M*. *persicae* and *A*. *fabae* was previously unknown.

**Results:**

In the laboratory bioassays, mortality of adult *M*. *persicae* and *A*. *fabae* caused by both *C*. *rosea* isolates varied according to conidial concentrations, with complete mortality occurring at 1 × 10^9^ conidia ml^−1^ 10 day post treatment. For *M*. *persicae*, LC_50_ values of AA80 and AA80 isolates were 3.6 × 10^6^ and 3.8 × 10^6^ conidia ml^−1^. For *A*. *fabae*, LC_50_ values of AA80 and AA80 isolates were 4.5 × 10^6^ and 4.35 × 10^6^ conidia ml^−1^. Infection by both fungal isolates at LC_50_ values reduced total fecundity of the treated aphids by 20% when compared to the untreated aphids.

**Conclusions:**

The results from laboratory bioassays showed that *C*. *rosea* has potential as a biological control agent of *M*. *persicae* and *A*. *fabae* which is crucial for ecofriendly biopesticide development. However, further field and greenhouse studies are required for mass production.

## Background

Aphids are one of the most important insect pests because they cause direct damage by feeding on crops and indirect damage as virus vectors [1]. In Iraq, the most economic damage aphid species attacking greenhouse crops are the green peach aphid, *Myzus persicae* Sulzer, and the bean aphid, *Aphis fabae* Scopoli (both Hemiptera: Aphididae) [2]. The two aphid species are polyphagous and capable of transmitting a large number of plant viruses [3]. The control of these insect pests relies mainly on the use of synthetic insecticides [4]. Unintended impacts of insecticides to natural enemy population and functions and risks to the environmental, as well as a high probability of resistance development encouraged the development of alternative strategies to control these aphid species [5].

Over the past 20 years, biological control programs anchored by the use of entomopathogenic fungi have been successful in managing aphid populations and damage on greenhouse crops, such as tomato, cucumber, and eggplant [6, 7, 2, 8]. Entomopathogenic fungi play an important role in aphid biological control programs because aphids have morphological, biological and ecological characteristics that make them susceptible to fungal pathogens that can cause epizootics [9].

*Clonostachys rosea* (formerly *Gliocladium roseum*) Schroers (Hypocreales: Bionectriaceae) is an important entomopathogenic fungal species [10]. It colonizes living plants as an endophyte, digests material in soil as a saprophyte, and also is known as a parasite of fungal pathogens and nematodes [11]. Zhang et al. [12] reported that *C*. *rosea* produces a wide range of volatile organic compounds that affect insects and other micro-organisms including fungi and bacteria. Toledo et al. [11] (2006) found that *C*. *rosea* isolates had high efficacy against two leafhopper pests, *Sonesimia grossa* Signoret and *Oncometopia tucumana* Schröder (Hemiptera: Cicadellidae) in Argentina. Anwar et al. [13] documented high efficacy of *C*. *rosea* isolates against fourth-instar and adult sweet potato whitefly, *Bemisia tabaci* Gennadius (Hemiptera: Aleyrodidae).

Reports on the efficacy of *C*. *rosea* against aphid species are not available. Such information may help integrate entomopathogenic fungi into an integrated pest management (IPM) program against *M*. *persicae* and *A*. *fabae* in greenhouse vegetable production systems. The aims of this research were to characterize two isolates of *C*. *rosea* originated from *M*. *persicae* population in Iraq and evaluate their efficacy against both *M*. *persicae* and *A*. *fabae* in laboratory bioassays. The sub-lethal effect of fungal infection on the fecundity of individual *M*. *persicae* and *A*. *fabae* adults was also investigated.

## Methods

### Sources of insects and fungi

Cultures of *M*. *persicae* and *A*. *fabae* were established with individuals collected initially from greenhouses at the Faculty of Agriculture, University of Kufa, Iraq, in 2018. The aphids were identified under a dissecting microscope, using the keys by Blackman and Eastop [14]. Cucumber, *Cucumis sativus* L. cultivar “Babylon” (Peto Seeds Company, Inc., Chicago, IL), were grown in the growth chamber for maintaining the aphid cultures and for the experiments. The aphid cultures were maintained in 45 × 45 × 45 cm cages at 23 ± 2 °C and 16:8 (L:D) h photoperiod for several generations. Plants were replaced every 2 weeks with uninfested 3–4-week-old plants.

Cultures of *C. rosea* were established by culturing dead *M*. *persicae* adults collected from greenhouses at the Faculty of Agriculture, University of Kufa (25 cadavers), and the Faculty of Agriculture, University of Basra, Iraq (25 cadavers). The aphid cadavers were surface sterilized with 1% sodium hypochlorite for 1 min, followed by washing with sterilized water and transferred to SDAY media. Plates, each with 10 cadavers, were incubated at 25 ± 1 °C for 7 day. A single tip from the mycelium were cut and sub-cultured on new plates for purification, and then stored at 4 °C as pure cultures. Morphological identification of fungal isolates were performed using Schroers [15] method. Aerial conidia were harvested from 10-day-old cultures by adding 12 ml 0.01% v/v Tween 80 (BDH Chemicals Ltd., Poole, UK) into the culture agar plates, then gently scraping the surface of the cultures with a sterile inoculating loop to dislodge the conidia from the surface. The conidial suspension was pipetted from the plate and filtered through three layers of sterile cheesecloth. The number of conidia in the suspension was determined using a hemocytometer (Neubauer Improved; Superior Marienfeld, Königshofen, Germany). The resulting suspension was diluted to the desired concentrations.

### Molecular identification and phylogenetic analysis

Genomic DNA of representative strains of *C. rosea* was extracted from freshly collected mycelium of 7-day-old cultures using a DNA extraction kit (Favorgen; Biotech Corp., Pingtung, Taiwan) following the manufacturer’s instructions. A spectrophotometer (NanoDrop 2000; Thermo Scientific, Waltham, Massachusetts) was used to quantify the extracted DNA and equalled with distilled water. The internal transcribed spacer (ITS) region of fungal isolates was amplified using polymerase chain reaction (PCR) with primer pairs ITS1 (TCCGTAGGTGAACCTGCGG and ITS4 (TCCTCCGCTTATTGATATGC) [16]. PCR was carried out in 20 μl reaction mix containing 1 μl ITS1 primer, 1 μl ITS4 primer, 12 μl GREENTaq DNA polymerase, 4 μl ddH2O, and 2 μl of template. PCR was performed with initial denaturation for 5 min at 98 °C, followed by 36 cycles of denaturation for 40 s at 94 °C, primer annealing for 40 s at 55 °C, and extension for 1 min at 72 °C. A final extension for 5 min at 72 °C was performed. The PCR products were run in electrophoresis-agarose gel at 1.5%, stained with 0.4 μg/ml ethidium bromide, and bands visualized with a UV illuminator (ULTRA TEC Manufacturing, Santa Ana, CA). PCR product was cleaned up with Exosape (Applied Biosystems, Foster, California) and sequenced by Macrogen Company (Seoul, South Korea).

Referenced sequences of similar regions from the genus *Clonostachys* were obtained from National Center for Biotechnology Information (NCBI) database [17]. Similar sequences were compared with sequences placed in GenBank databases by using of the Basic Local Alignment Search Tool (BLAST) [17]. Related sequences were aligned using DNA Baser software and a phylogenetic tree was built with Geneious software version 10.2.3 [18].

### Virulence bioassays

This experiment was conducted to evaluate the virulence of AA80 and AA82 isolates against adults of *M*. *persicae* and *A*. *fabae*. In order to obtain a uniform age (1–2-day-old) of adult *M*. *persicae* and *A*. *fabae* for use in all experiments, 20 adults of each species were transferred from the stock cultures onto each 4-week-old cucumber plants, isolated in 3-cm-diameter clip-cages, and allowed to produce nymphs for 1 day. After the removal of the adults and excess nymphs, 25 nymphs were left on each plant and allowed to develop for nine additional days before treatment in a growth chamber (Binder Ltd., Suffolk, UK) at 20 ± 2 °C, 75 ± 2% RH and a photoperiod of 16:8 h (L:D). The clip cages were removed before the treatment.

Two milliliters of conidia suspensions (1 × 10^5^, 1 × 10^6^, 1 × 10^7^, 1 × 10^8^, or 1 × 10^9^ conidia ml^−1^) of either *C*. *rosea* isolate AA82 or isolate AA80 were applied onto each infested 4-week-old potted cucumber plant using a hand-held sprayer (Ampulla, Hyde, UK). Plants assigned to the control treatment were sprayed with 0.02% aqueous Tween 80 solution. Eight replicates were carried out for the control and each concentration of each fungal isolate against either *M*. *persicae* or *A*. *fabae*. Plants were air-dried on bench top at room temperature for 1 h. The treated plants were then transferred to the growth chamber maintained at 26 ± 1 °C, ≥ 70% RH and a photoperiod of 16:8 (L:D) h. To prevent aphids from moving between treated plants, the aphids were kept in the 3-cm-diameter clip-cages post-spraying. Aphid mortality was recorded at 1, 3, 5, 7, and 10 day after application. Dead aphids were surface sterilized by rinsing twice with 70% ethanol for 30 s and then with sterilized distilled water, after which they were placed on water agar (3 g of agar per L of water) in Petri dishes for 5 day to confirm infection by *C*. *rosea* [19]. A cadaver was regarded as dead from infection by these fungi if the conidia were recovered from the cadaver. Median lethal concentration (LC_50_) and median lethal time (LT_50_) were calculated.

### Effect of fungal infection on aphid fecundity

This experiment was conducted to determine the effect *C*. *rosea* infection on nymph production by *M*. *persicae* and *A*. *fabae* adults. The method described above was used to obtain uniform age of adults of either *M*. *persicae* or *A*. *fabae*. The LC_50_ value of each isolate was sprayed once onto infested leaves of cucumber plants (2 ml per leaf) each harboring 20 adult *M*. *persicae* or *A*. *fabae*. Plants assigned to control treatment were sprayed with 0.02% Tween 80 solution. After treatment, the plants were kept in the laboratory at room temperature for 1 h to dry, and then transferred to a growth chamber maintained at the same environmental conditions described above. After 1 day, a treated aphid (5 aphids from each plant leaf) was selected randomly and transferred with a fine camel hairbrush onto an uninfested cucumber plant and isolated with a new 3-cm-diameter clip-cage (2 clip-cages per plant). A total of 25 individuals of each aphid species were isolated in this manner for each fungal isolate treatment. Plants were then transferred to the same growth chamber maintained at the same conditions described above. Each leaf clip-cage was carefully opened and inspected daily until the death of the aphids and the number of nymphs produced by each individual was recorded.

### Statistical analysis

Statistical analysis was performed using GenStat software (VSN International 2016). Cumulative mortality was corrected for control mortality by removing the number of dead aphids in the control data from the corresponding number. Normal distribution of data was assessed using the Shapiro-Wilk test. Mortalities were logit transformed when necessary to meet the assumption of normality. LC_50_, LT_50_, and 95% confidence limit of each *C*. *rosea* isolate against each aphid species were calculated by probit regression analysis. Two-factor repeated measure ANOVA was used to determine the effect of fungal isolate and aphid species on aphid mortality. The effect of fungal infection on aphid fecundity was analyzed separately for each aphid species using one-way measure ANOVA. Mean comparisons were performed using Turkey’s test at *P* = 0.05.

## Results

### Characterization and phylogeny of *C. rosea* isolates

**Fig. 1:**
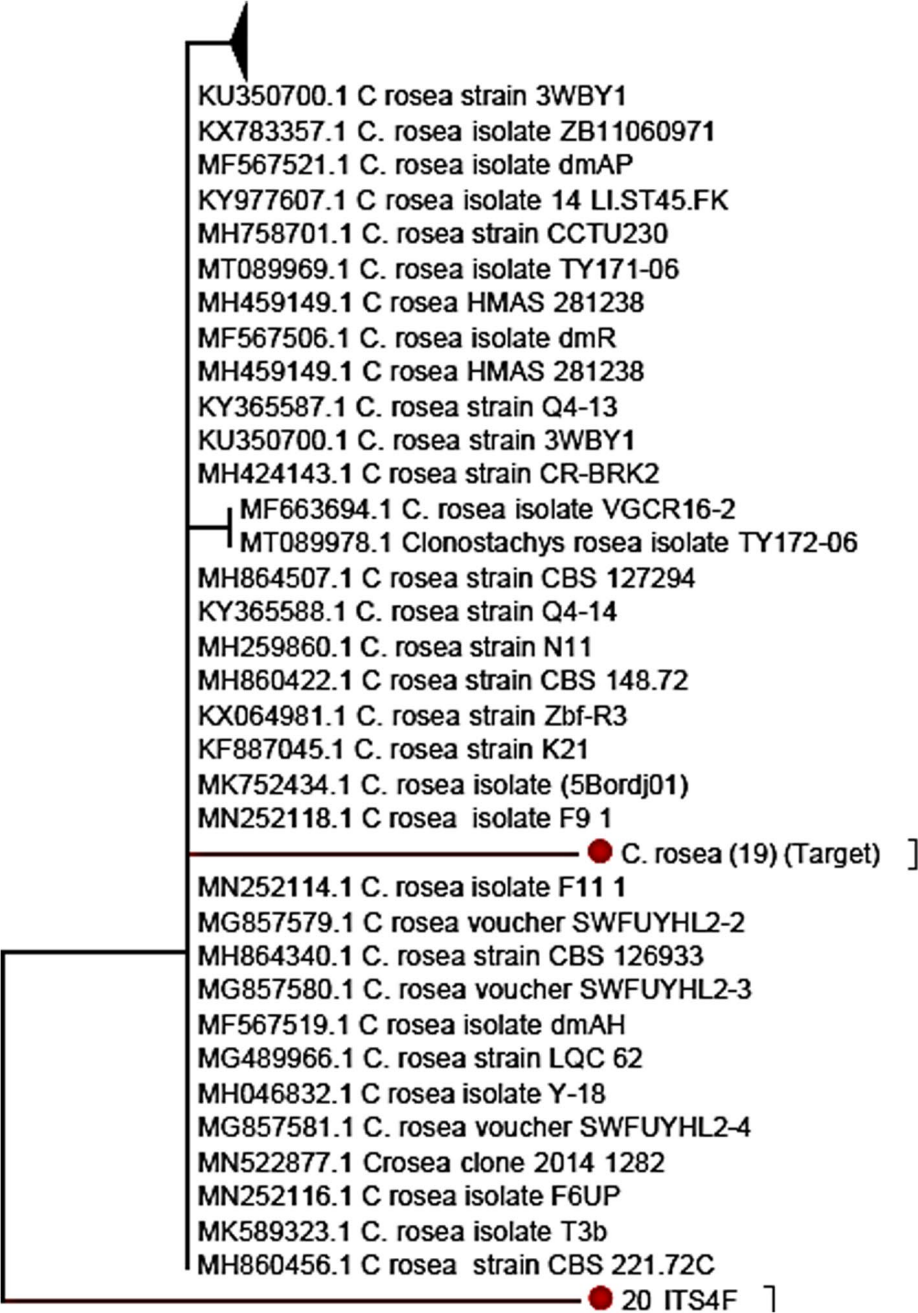
Phylogenetic tree showing relationships of new submitted isolates of *Clonostachys rosea* (19-AA80, 20-AA82) based on ITS region NCBI accession number MT366561 and MT366214, respectively

Sequences obtained in this study and compared against sequences in NCBI databases had 99% similarity with other *C. rosea* entries. Two isolates, labeled AA80 (19) and AA82 (20), were characterized in this study. The isolates’ sequences were submitted for the first time to the NCBI and registered under the Accession Number MT366561 (AA80) and MT366214 (AA82). The sequences of AA80 and AA82 different at six SNPs at 15, 16, 17, 325, 404, and 488. The neighbor-joining tree (NJ tree) indicated that the two isolates were classified into different clades within *Clonostachys* (Fig. 1). The first isolate, AA80 (MT366561), was placed in a clade with maximum similarity to *C. rosea* (Accession Number KF887045). The second isolate, AA82 (MT366214), was placed in a clade with maximum similarity of *C. rosea* (Accession Number MH860456).

### Virulence bioassay

**Fig. 2:**
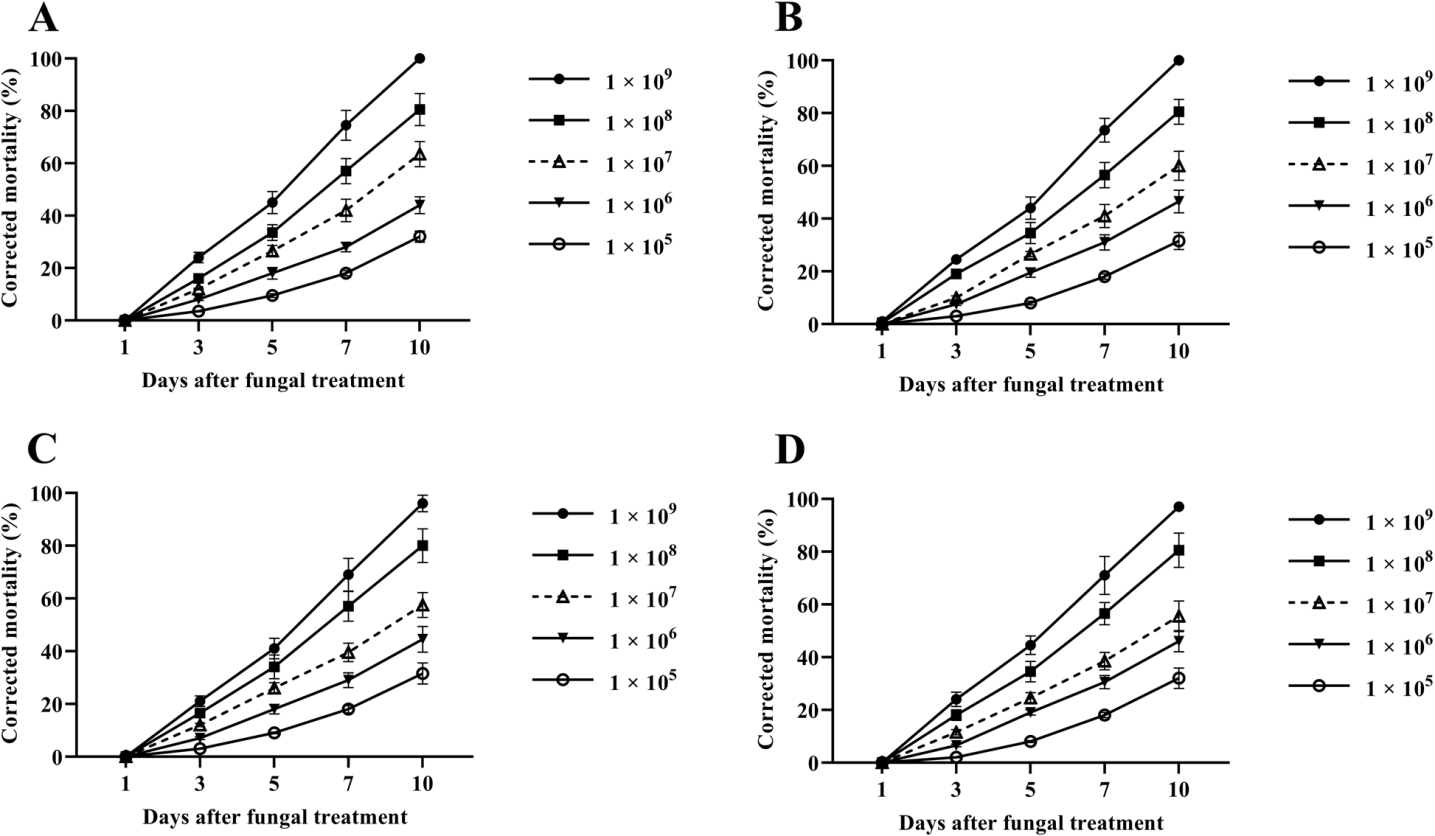
The effect of conidial concentration and period of time after treatment on the corrected mortalities of *M*. *persicae* treated with *C*. *rosea* isolate AA80 (**A**) and isolate AA82 (**B**) and *A*. *fabae* treated with *C*. *rosea* isolate AA80 (**C**) and isolate AA82 (**D**)

Isolate AA80 and AA82 exhibited similar pathogenicity against adult *M*. *persicae* and *A*. *fabae* (aphid species: *F*_1, 159_ = 0.58, *P* = 0.453; fungal isolate: *F*_1, 159_ = 0.39, *P* = 0.535). Cumulative mortality of *C*. *rosea* isolates were dose-dependent and mortality obtained with both isolates at the highest dose of 1 × 10^9^ conidia ml^−1^ was 100% for *M*. *persicae* and 97% (AA80) and 96% (AA82) for *A. fabae* at 10 d post-treatment (Fig. 2).

For *M*. *persicae*, LC_50_ of isolate AA80 was 3.6 × 10^6^ conidia ml^−1^ and LT_50_ was 6.1 day. LC_50_ and LT_50_ values of isolate AA82 were not significantly different compared to isolate AA80 (3.8 × 10^6^ conidia ml^−1^ and 6.3 day) (slope = 1.35, χ^2^ = 1.06, *P* = 0.64). For *A*. *fabae*, LC_50_ value of isolate AA80 was 4.5 × 10^6^ conidia ml^−1^ and LT_50_ was 6.2 day. LC_50_ and LT_50_ values of isolate AA82 were not significantly different compared to isolate AA80 (4.35 × 10^6^ conidia ml^−1^ and 6.1 day) (slope = 0.96, χ^2^ = 1.84, *P* = 0.81).

### Effect of fungal infection on aphid fecundity

**Fig. 3:**
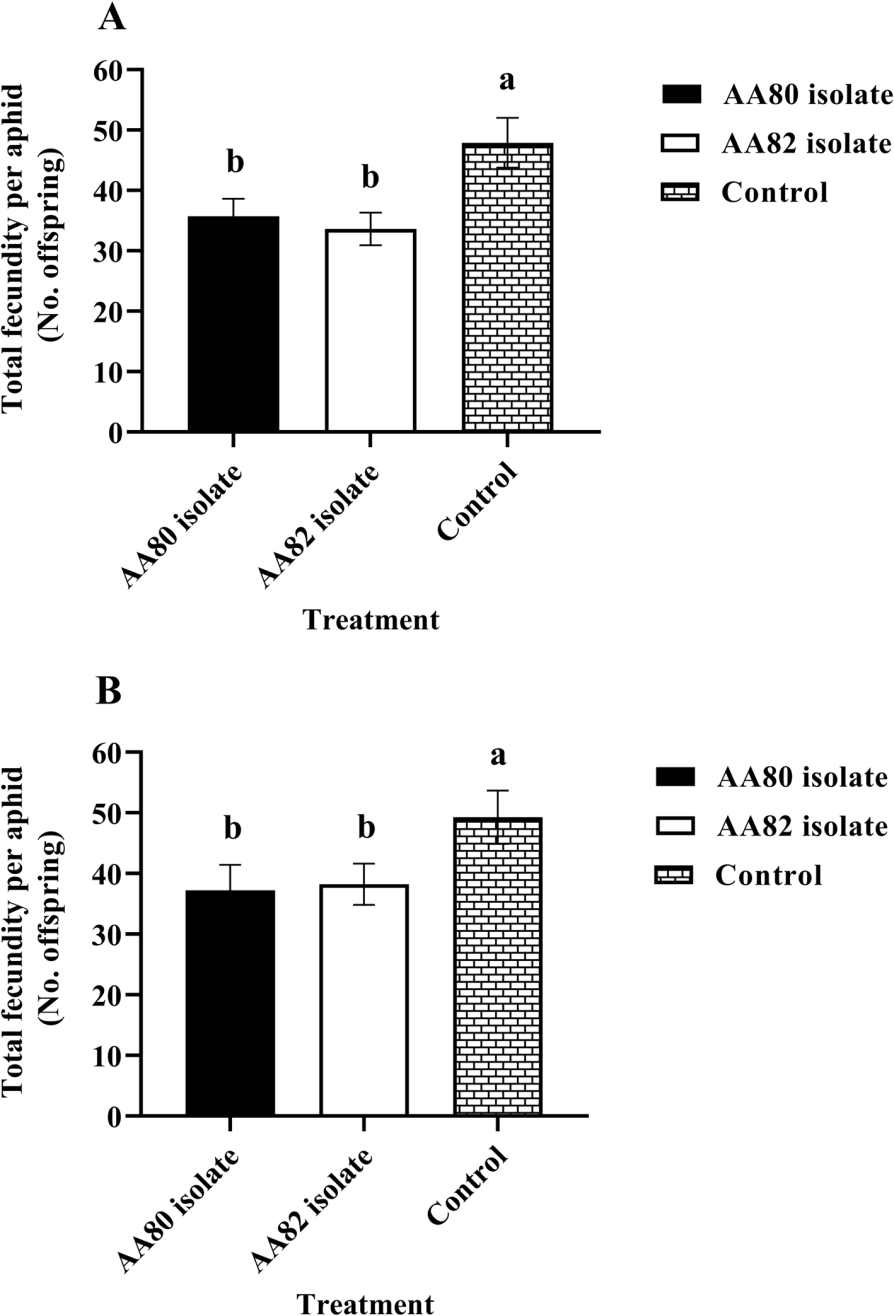
Mean fecundity of *M*. *persicae* (**A**) and *A*. *fabae* (**B**) after treatment with 0.01% v/v Tween 80 solution (control) and two isolates of *C*. *rosea*. Bars topped with different letters are significantly different according to Turkey’s test at *P* = 0.05

The total fecundity of both *M*. *persicae* and *A*. *fabae* adults were affected by fungal infection (*M*. *persicae*: *F*_1, 134_ = 5.77, *P* < 0.001; *A*. *fabae*: *F*_1, 134_ = 5.84, *P* < 0.001). *Myzus persicae* exposed to Tween 80 solution (control) produced 30% more offspring than aphids exposed to isolate AA80 and AA82 (Fig. 3A). *Aphis fabae* exposed to Tween 80 solution produced 32.2% more offspring than aphids exposed to isolate AA80 and AA82 (Fig. 3B).

## Discussion

Although *C*. *rosea* can be isolated from many habitats, including different types of soil and decaying plant materials, it rarely has been isolated from dead insects. Both *C*. *rosea* strains originated from *M*. *persicae* in this study showed characteristics similar to those observed by Anwar et al. [13]. Comparison to sequences in the NCBI database indicated that AA80 and AA82 are considerably close to other groups of the genus *C. rosea*. Molecular approaches are significant tools in simplifying the complex issues of genus and species taxonomy. Although both isolates are unique from the other previously identified isolates, the isolates showed less genetic variation among each other. This might lead to hypothesize that sample location and host type may not influence the genetic makeup of the isolates recovered.

The virulence bioassay is an important for the success of biological control methods toward aphids and other insect pests [20] as a result of the virulence of EPF strains might be varied based on their geographical and ecological exposures, genetic variation, or adaptive attributes of enzyme-coding genes. Therefore, we have investigated two strains of *C*. *rosea* from the different sources of local environment to obtain effective candidates for aphid control Both *C*. *rosea* strains showed promising mortality rates against adult *M*. *persicae* and *A*. *fabae*, compared with the commercially formulated product (Naturalis-L) that scored about 65% mortality rates against *M*. *persicae* and *A*. *fabae* at a concentration of 1 × 10^7^ conidia·ml^−1^ in Mohammed et al. [2]. This could infer that locally screened isolates might be pathogenically effective than commercialized ones, and geographic origin of isolates may determine their pathogenicity. These results confirmed the findings of Mohammed et al. [21] who demonstrated that both the mortality caused by both *C*. *rosea* isolates against adult stages of *Trogoderma granarium*, *Tribolium castaneum*, and *Callosobruchus maculatus* ranged from 70.7 to 75.7% at a concentration of 1× 10^8^ conidia ml^−1^. Contrary to our findings, Anwar et al. [13] found that the mortality caused by different isolates of *C*. *rosea* against adults and nymphs of *B. tabaci* were 23.5% and 50.4%, respectively, 6 days post-treatment. In addition, Toledo et al. [11] reported that the mortality obtained by *C*. *rosea* against *O. tucumana* was 82.5% at 14 day post-treatment. This may be due to the use of different insect hosts and/or variation of *C*. *rosea* isolates in the quantity of produced protease which play a role in the rapid penetration of insect cuticle and that reflects their virulence [22]. This could infer that geographic origin of isolates may determine their pathogenicity.

It is important for EPF to determine the optimal conidial concentration and medium lethal time to reduce the overall cost of insect pest control and achieving a high level of control. The LC_50_ values for *M*. *persicae* and *A*. *fabae* exposed to both *C*. *rosea* isolates were not significantly different, which are ranged between 3.6 × 10^6^ and 4.5 × 10^6^ conidia ml^−1^. However, Anwar et al. [13] found that the LC_50_ of *C*. *rosea* isolates against fourth instar nymph and adult stages of *B*. *tabaci* were much higher compared with the findings of the current study. The variation in LC_50_ values could be explained by differences in the target insect species, application method and/or environmental conditions. Both *C*. *rosea* isolates scored a LT_50_ values for *M*. *persicae* and *A*. *fabae* ranged between 6.1 and 6.2 days at a concentration of 1 × 10^7^ conidia ml^−1^. The LT_50_ values obtained are considerably longer than those achieved by Mohammed et al. [21] who found that both *C*. *rosea* isolates had the LT_50_ values ranged from 4.79 to 4.95 days at a conidial concentration of 1 × 10^8^ conidia ml^−1^ against coleopteran stored product insect pests. Usually, when the conidial concentration of EPF increases, median lethal time decreases concurrently, which is comparable with other findings.

Isolate AA80 and AA82 significantly reduced the total fecundity of *M. persicae* and *A. fabae*. Fungal infection increased aphid mortality, which led to a reduced reproductive duration [19]. The negative effect could be attributed to decrease the daily rate of nymph production of fungus-treated aphids [23]. Infection by EPF can also result in reduced food intake in aphids, which is probably a contributing factor to the reduction in reproductive rate [24]. Similar to our findings, Wang & Knudsen [25] and Kim [26] reported that the total fecundity was significantly decreased due to an increasing level of aphid mortality. In addition, Shrestha et al. [27] found a negative effect on the daily rate of nymph production of *Nasonovia ribisnigri* Mosley (Hemiptera: Aphididae) treated with either high, medium, or low conidial concentrations of *Beauveria bassiana* Vuill (Hypocreales: Cordycipitaceae).

## Conclusion

In conclusion, molecular analysis of new *C*. *rosea* isolates showed that they are unique from the other previously identified isolates, but both isolates showed less genetic variation among each other. The laboratory experiments reported here indicate that new *C*. *rosea* isolates _ could be an appropriate biological control agent for controlling both *M*. *persicae* and *A*. *fabae*. Fungal infection does not have a physiological effect on the developing progeny. However, further studies to confirm the efficacy of these new *C*. *rosea* isolates under greenhouse and field conditions will be required for mass production. In addition, further investigation to determine the efficacy of *C*. *rosea* isolates against other important insect pests are required.

## Data Availability

All data generated or analyzed during this study are included in this published article.
